# Morphological and metabolic determinants of nonalcoholic fatty liver disease in obese youth: a pilot study

**DOI:** 10.1186/1756-0500-6-89

**Published:** 2013-03-09

**Authors:** Paula A Monteiro, Jorge Mota, Loreana S Silveira, Suziane U Cayres, Bárbara de Moura M Antunes, Romulo Araujo Fernandes, Ismael F Freitas

**Affiliations:** 1 Department of Physiotherapy, University Estadual Paulista, Campus of Presidente Prudente, 160, Redentore Gonfiantine St., São Paulo, Vila São Francisco, Zip code 05351020, Brazil; 2 Research Centre in Physical Activity, Health and Leisure, Faculty of Sports – University of Porto, Porto, Portugal; 3 Department of Physical Education, University Estadual Paulista, Campus of Presidente Prudente, São Paulo, Brazil; 4 Department of Physical Education, University Estadual Paulista, Campus of Rio Claro, São Paulo, Brazil

**Keywords:** Adolescents, Children, Nonalcoholic fatty liver disease, Obesity

## Abstract

**Background:**

Nonalcoholic fatty liver disease (NAFLD) related to obesity has been rising in the last decades, though the morphological and metabolic determinants are remain unclear in children. The aim of this study was to analyze the morphological determinants and metabolic abnormalities in obese children and adolescents, classified either as with (P-NAFLD) or without (N-NAFLD). The sample comprised 190 individuals, aged 6 to 16 years-old, assigned into one of 4 groups according to sex and presence or absence of NAFLD. Obesity was obtained according to body mass index (BMI) cut-points. Body composition variables was estimated by Dual-Energy X-ray Absorptiometry (DEXA). Total cholesterol (TC), triglycerides (TG), HDL-cholesterol (HDL-C), LDL-cholesterol (LDL-C), insulin, fasting glucose (FG) and blood pressure were also analyzed. The diagnosis of NAFLD, as well as the measurement of intra-abdominal fat tissue thickness (IAF) and subcutaneous abdominal fat tissue thickness (SCF), was carried-out by ultrasound.

**Results:**

Males and females belonging to P-NAFLD group showed, respectively, higher TFM and IAF. When data were adjusted for sex, age and total fat mass, those in P-NAFLD showed statistically higher IAF, TFM and TG.

**Conclusion:**

Our study showed that obese youngsters who were assigned to P-NAFLD group were twice as likely to present higher concentration of triglycerides, higher levels of trunk fat, as well as intra-abdominal fat compared to their N-NAFLD counterparts even after adjustments for sex, age, pubertal stage and total body fat mass.

## Background

Nonalcoholic fatty liver disease (NAFLD) or liver steatosis is characterized by a pathological fat accumulation in the liver, ranging from simple steatosis to non-alcoholic steatohepatitis, advanced fibrosis, cirrhosis and liver cancer [1].

Although the prevalence of NAFLD is not well known, it is the most common form of pediatric liver disease [2]. Fat accumulation in the liver, mainly in the triglycerides form, can range from5 to 10% of the liver weight [2,3] and studies have shown a strong association between obesity and NAFLD [4]. With increasing obesity rates globally, NAFLD is considered a growing public health concern [5]. The pathogenesis of NAFLD is complex and comprises multiple factors such as genetic predisposition, life style, visceral adiposity, insulin resistance, ethnicity, age and sex [6,7]. Some studies indicate that the histopathological signs of steatosis can appear between 11 and 13 years, for both sexes. This timing suggests that puberty may play an important role inthe development of this pathology [8] due to the increased production of sex hormones and insulin resistance, which might be crucial to NAFLD genesis [9].

Although obese individuals are more prone to present NAFLD, data regarding the differences in clinical manifestation and related metabolic abnormalities in those with and without NAFLD diagnosis is scarce. To the best of our knowledge there is only one study in Brazil that has addressed this issue so far, having examined the body composition and metabolic profile in young obese with NAFLD diagnosis in post-apex pubertal stage [4]. However, the data were not analyzed according to maturational stage and did not indicate whether or not fat distribution and cardio-metabolic profile are associated NAFLD. To fill this gap in the literature, our study aimed to analyze the association between NAFLD and morphological determinants in a sample of Brazilian obese youngsters.

## Methods

### Participants and setting

Data of 190 obese children and adolescents, aged 6 to 16 years old, were collected during the second semester of 2010. The participants were invited, through media advertisement, to participate in a community-based program. This program aimed to treat obesity focused on enhancing exercise and nutritional orientation and was carried out at facilities of the Universidade Estadual Paulista - Campus of Presidente Prudente (FCT/UNESP), São Paulo, Brazil.

The inclusion criteria were: (a) to be classified as obese according to the reference values proposed by Cole et al. [10], (b) aged between 6 and 16 years at the first evaluation date, and (c) participants and parents/guardians signed a written informed consent form for the participation in the program.

The Ethical Committee of FCT/UNESP approved this study (Protocol number 07/2009).

### Anthropometry

Body weight (BW) was measured with an electronic scale (precision 0.1 kg [Filizzola PL 150, Filizzola Ltda]) and the height (H) with a wall-mounted stadiometer (precision 0.1 cm [Sanny^®^, São Paulo, Brazil]). Both measurements were carried out with participants wearing light clothing and no shoes. Body mass index (BMI) was then calculated as kg/m^2^. All anthropometric measurements were performed by trained researchers, according to standardized techniques [11].

### Dual Energy X-Ray Absorptiometry

Body composition was estimated by a Dual-energy X-ray absorptiometry (DEXA) scanner (Lunar DPX-NT; General Electric Healthcare, Little Chalfont, Buckinghamshire), with software version 4.7. The method estimated the body composition by fractionating the body into three anatomical compartments: fat-free mass (FFM), fat mass (FM) and bone mineral content. The assessment was carried out in approximately 15 minutes, and the subjects remained still and in a supine position throughout the scan, wearing light clothing while lying flat on their back with arms by the side. The results were expressed in kilograms of FFM and FM. The absolute (kg) and relative (%) values from trunk fat (%TF) were also assessed by DEXA. All DEXA measurements were carried-out at the University laboratory in a controlled temperature room. Each morning, before the beginning of the measurements, the DEXA was calibrated by the same researcher, according to the references provided by the manufacturer.

### Stages of puberty

The stage of puberty was self-assessed by the participants aged older than 10 years. A standardized series of drawings was given to the subjects to assess their own pubertal development. Girls received drawings of the five stages of Tanner breast and female pubic hair development with appropriate descriptions accompanying the drawings. Boys were given line drawings of boys showing the five Tanner stages of pubic hair development, with appropriate written descriptions. Participants were asked to select the drawing of the stage that best indicated his other own development. All procedures were done as described by Marshall & Tanner [12]. The results were placed by each subject in a locked box with only their subject id as an identifier, so as to guarantee the integrity and anonymity of the subjects. Only the main researcher had access to those questionnaires.

The breast stages for girls and genitalia stages for boys were chosen to classify the sexual maturation status because they are recommended by a World Health Organization Expert Committee as indicators of sexual maturation for international use [13]. The original five pubertal stages were grouped in three new groups: participants aged under 10 years and those who answered stage 1, were grouped in group 1; those who answered stages 2 and 3, were grouped in group 2; and those who answered stages 4 and 5, were grouped in group 3.

### Ultrasound

The ultrasound equipment (Toshiba Aplio Model Tochigi-ken, Japan) was used by a trained radiologist to assess the level of fat accumulation and the morphology of the liver. Finally the NAFLD diagnosis was made by a hepatologist. The NAFLD was graded semi qualitatively as described by Shannon [14]: Score 0 is absence of steatosis, defined as normal liver echo texture; Score 1 is mild steatosis, defined as slight and diffuse increase in fine parenchymal echoes, normal visualization of diaphragm and portal vein borders; Score 2 is moderate steatosis, defined as a diffuse increase in fine echoes with slightly impaired visualization of diaphragm and portal vein borders and; Score 3 is severe steatosis, defined as fine echoes with poor or no visualization of diaphragm, portal vein borders and posterior portion of the right lobe. The participants were labeled as “without NAFLD” (N-NAFLD) and those with any of the above mentioned grade of fatty infiltration in the liver were labeled as “with NAFLD” (P-NAFLD).

The ultrasound is an image evaluation that provides diagnosis of NAFLD and measures, in centimeters, the length of intra-abdominal fat (IAF) and subcutaneous fat (SCF).

### Blood samples

A 12-hour fasting blood sample collection was taken and analyzed by a private laboratory located in the city of Presidente Prudente, Brazil. Samples were collected in vacuum tubes containing gel with anticoagulant. Then the blood was centrifuged for 10 minutes at 3,000 rpm. To measure total cholesterol (TC), triglyceride (TG), HDL-cholesterol (HDL-C), LDL-cholesterol (LDL-C) and glucose (GL), an enzymatic colorimetric kit processed in a unit Autohumalyzer A5 was used [15]. Insulin was analyzed using the RayBio^®^ Human Insulin ELISA Kit, following the Manual Revised Nov 23, 2009.

### Blood pressure

An automated digital blood pressure monitor (Omron Healthcare, Inc., Intellisense, modelo HEM 742 INT, Bannockburn, Illinois, USA) was used for the measurement of systolic (SBP) and diastolic (DBP) blood pressure. This equipment was previously validated for pediatric population [16]. After fifteen minutes of resting in a lying-down position, two measures were taken on the right arm, with a two minute interval between them. The mean value was used for statistical analysis.

### Diagnosis of metabolic syndrome

For diagnosis of Metabolic Syndrome (MS), we adopted the cut-point proposed by the World Health Organization (WHO) [17] for adolescents. The MS was diagnosed in subjects who presented three or more of the following risk factors: Obesity (BMI > percentile 95 according Must [17]); Glucose Homeostasis (prepubertal hyperinsulinemia >15 mU/L; [18] [stage 1 Tanner] pubertal>30 mU/L [19]; [stages 2–4 Tanner] pospubertal ≥20 mU/L [stage 5 Tanner]; Fasting Glucose ≥6.1mM/L and Impaired Glucose Tolerance: 120min ≥ 7.8mM/L), Elevated Blood Pressure (SBP > perc95 for age, sex and height proposed by NHBPEP [19]). Dyslipidemia (Triglycerides >105mg/dL for children< 10 years and >136mg/dL for children ≥ 10 years, HDL-C <35 mg/dL and Total Cholesterol > percentile 95).

### Data analysis

Descriptive data are shown as means and standard deviation. One-way analysis of variance (ANOVA) was applied to analyze the differences in body composition, blood pressure and biochemical variables between males and females within NAFLD groups. A multivariate analysis was used to compare all variables within NAFLD group as dependent variable. Data were adjusted for sex, age, pubertal stage and total fat mass. The relative risk (odds ratio) and 95% confidence interval were calculated by binary logistic regression. The statistical significance was set at 5% for all the analyses and the calculations were conducted using SPSS, version 17.0 (SPSS Inc. Chicago. IL).

## Results

Table 1 shows general characteristics and comparisons of morphological and metabolic risk factors, according to sex, as well aspresence and absence of NAFLD. Among children and adolescents evaluated in this study, 18.7% (n = 35) had metabolic syndrome and 25.7% (n = 48) had DGNAF. Of youth with DGNAF, 9.1% (n = 17) also had syndrome metabolic. The comparisons showed statistical differences in the BW (p=0.003), FM (p=0.042), FFM (p<0.001) and SBP (p=0.039) between males and females, respectively, with and without NAFLD; BMI (p=0.001) between males with and without NAFLD; FG (p<0.001) between females with and without NAFLD, and in the TFM (p=0.002) between males with and both sexes without NAFLD.

**Table 1:** General characteristics and comparison of morphological and metabolic syndrome components of obese adolescents with and without nonalcoholic fatty liver disease

	**Male N-NAFLD**	**Male P-NAFLD**	**Female N-NAFLD**	**Female P-NAFLD**	
	**Mean (SD)**	**Mean (SD)**	**Mean (SD)**	**Mean (SD)**	***p***
Age (years)	10.8(2.6)	121(2.5)	11.2(2.7)	10.7(2.7)	0.063
Weight (kg)	67.8(20.4)^ab^	78.9(22.4)^a^	63.4(15.0)^b^	66.2(24.7)^ab^	0.003
Height (cm)	151.1(13.8)	155.8(13.1)	149.9(11.3)	149.8(13.5)	0.170
BMI (kg/m^2^)	29.0(4.7)^a^	31.8(4.9)^b^	27.8(3.7)^ac^	28.6(5.8)^abc^	0.000
GL (mg/dl)	82.2(5.8)	82.7(6.2)	80.9(6.3)	80.8(7.5)	0.399
TG (mg/dl)	113.9(49.4)	138.8(78.7)	111.0(42.4)	125.7(43.0)	0.073
TC (mg/dl)	167.0(35.5)	168.3(33.2)	163.6(30.9)	166.5(23.4	0.883
HDL-C (mg/dl)	43.4(10.3)	40.9(11.3)	44.2(9.5)	41.4(11.0)	0.418
LDL-C (mg/dl)	100.7(31.8)	99.5(32.7)	97.1(27.3)	99.9(18.1)	0.909
SCF (cm)	3.0(1.2)	3.4(1.1)	3.0(0.9)	2.9(1.0)	0.316
IAF (cm)	4.6(1.5)^a^	5.2(2.0)^a^	3.7(1.1)^b^	4.9(1.1)^a^	0.000
%TF	44.3(5.9)	45.2(4.9)	46.2(4.7)	45.8(4.9)	0.220
FM (Kg)	30.1(10.8)^ab^	35.3(11.9)^a^	29.0(9.1)^b^	30.1(118)^ab^	0.042
FFM (kg)	34.8(9.9)^ab^	40.1(11.5)^a^	30.8(6.6)^b^	33.4(13.0)^ab^	0.000
TFM (kg)	13.5(5.2)^a^	17.0(5.9)^b^	13.0(4.1)^ac^	14.3(5.7)^abc^	0.002
SBP (mmHg)	119.0(14.2)^ab^	124.3(13.6)^a^	116.2(10.3)^b^	119.1(12.5)^ab^	0.039
DBP (mmHg)	70.0(7.8)	72.4(8.9)	69.1(6.6)	71.1(5.6)	0.222
Insulin (μUI/mL)	61.6(51.4)	56.2(59.8)	42.1(54.6)	65.8(83.1)	0.436

N-NAFLD absence of nonalcoholic fatty liver disease; P-NAFLD presence of nonalcoholic fatty liver disease; SD= standard deviation; BMI= Body Mass Index; GL = glucose; TG = triglycerides; TC = total cholesterol; HDL-C = high density lipoprotein-cholesterol; LDL-C = low density lipoprotein-cholesterol; SCF = Subcutaneous abdominal fat tissue thickness; IAF = Intra-abdominal fat tissue thickness; %TF = Percentage of trunk fat; FM= Fat mass; FFM= Fat-free mass; TFM= Trunk fat mass; SBP= systolic blood pressure; DBP= diastolic blood pressure; p<0.005; superscript letter means statistical difference between groups.

In Table 2 illustrates the multivariate analysis findings. After adjustments for sex, age, pubertal stage and total body fat mass, statistically significant differences were observed in TG (p=0.036), TFM (p=0.001) and IAF (P=0.002) for both NAFLD groups.

**Table 2:** **Comparisons of body composition and metabolic syndrome components of obese adolescents with (presence) and without** (**absence**) **nonalcoholic fatty liver disease**, **adjusted by sex**, **age**, **pubertal stage and total body fat mass**

		**Mean (SE)**	**CI**		**f**	***p***
TFM (kg)	Presence	13.5(0.1)	13.3	13.8	11.49	0.001
	Absence	14.5(0.2)	14.0	151		
SCF (cm)	Presence	3.1(0.0)	3.0	3.2	0.20	0.648
	Absence	3.0(0.1)	2.8	3.3		
IAF (cm)	Presence	4.0(0.1)	3.8	4.3	10.03	0.002
	Absence	4.8(0.2)	4.4	5.2		
GL (mg/dl)	Presence	81.7(0.5)	80.6	828	0.04	0.839
	Absence	81.5(0.9)	795	83.5		
TG (mg/dl)	Presence	114.6(4.8)	105.0	1242	4.48	0.036
	Absence	135.6(8.6)	118.6	152.6		
TC (mg/dl)	Presence	163.8(2.8)	158.2	169.3	0.00	0.983
	Absence	164.0(4.9)	154.2	173.8		
HDL-C (mg/dl)	Presence	43.6(0.9)	41.8	45.4	2.12	0.147
	Absence	40.8(1.6)	37.6	44.0		
LDL-C (mg/dl)	Presence	97.2(2.5)	92.1	102.3	0.07	0.792
	Absence	95.9(4.5)	87.0	104.9		
SBP (mmHg)	Presence	118.2(1.0)	116.1	120.3	1.14	0.286
	Absence	120.8(1.8)	117.1	124.4		
DBP (mmHg)	Presence	69.8(0.6)	68.5	71.2	1.51	0.220
	Absence	71.6(1.2)	69.2	73.9		
Insulin (μUI/mL)	Presence	47.6(5.7)	36.2	59.0	1.62	0.205
	Absence	62.4(10.2)	42.3	82.6		

SE= Standard error; CI= Confidence Interval; p<0.005; TFM= trunk fat mass; SCF= Subcutaneous abdominal fat tissue thickness; IAF= Intra-abdominal fat tissue thickness; GL= glucose; TG= triglycerides; TC= total cholesterol; HDL-C= high density lipoprotein; LDL-C= low density lipoprotein; SBP= systolic blood pressure; DBP= diastolic blood pressure; 0= Absence of nonalcoholic Fat Liver Disease; 1= Presence of nonalcoholic Fat Liver Disease.

Table 3 shows the findings of regression analysis in which only those variables that showed statistical significance were incorporated into the model. Males of P-NAFLD group were more likely to have higher levels of %TF above median value (46.62%) while P-NAFLD females were more likely to show IAF values above median (3.75cm).

**Table 3:** Odds ratio of body composition and ultrasound analysis and of children and adolescents with and without nonalcoholic fatty liver disease

		**Dependent variable: Nonalcoholic fatty liver disease**		
**Male (Median)**		**OR**	**(OR**_**95%CI**_**)**	***P***
%TF (46.62%)	Below	1	---	---
	Above	2.66	(1.06 – 6.69)	0.005
Female (median)		OR	(OR_95%CI_)	*P*
IAF (3.75 cm)	Below	1	---	---
	Above	3.09	(1.01 – 9.48)	0.048

OR= odds ratio; 95%CI= 95% confidence interval; %TF= percentage of trunck fat; LL= Left lobe of liver; IAF=Intra-abdominal fat tissue thickness; IAF+SCF=Intra-abdominal plus Subcutaneous abdominal fat tissue thickness.

## Discussion

The purpose of the present study was to analyze the morphological determinants and metabolic abnormalities in obese children and adolescents, classified according to positive or negative diagnosis to NAFLD.

The main results of the present study are that gender differences seem to be associated with NAFLD in obese youth. Indeed, P-NAFLD males were more likely to have higher levels of abdominal fat mass, while P-NAFLD females were more likely to show higher intra-abdominal fat mass. Our data are noteworthy. Indeed, the relationship between obesity and NAFLD in children was also observed in a recent study [7]. Additionally, obese individuals are often afflicted with comorbidities caused by obesity, such as NAFLD [20,21], that can evolve to more damaging stages as cirrhosis and liver carcinoma [7].

In pediatric populations, previous studies have reported that NAFLD is related to risk factors such as: whole body and central obesity [6,22], insulin resistance and type II diabetes [23]. Studies also suggest that children who enter puberty with excessive fat and/or present genetic basis are more likely to develop NAFLD [8,24]. Nonetheless, the amount of fat accumulated in the deep abdominal region seems to be a key-factor in NAFLD diagnosis. Furthermore, the profound endocrine, metabolic and morphologic changes that occurred over time, particularly in adolescence might explain the gender differences found in the occurrence of NAFLD [25].

Indeed, despite the effect of estrogen hormone, which stimulates accumulation of fat in the hip area and contributes towards the cardiovascular risk events decrease in women [26-28], as well as provides a protective effect on the accumulation of fat in the liver, our results indicate that a small increase in the amount of IAF in girls is associated with P-NAFLD, independently of total fat mass, sex, pubertal stage and age.

These findings are consistent with those obtained in previous studies [29,30], suggesting that visceral adiposity is the best variable for predicting the fat accumulation in the liver, with a further possible explanation for the differences between sex, being related to sex hormones during puberty [28].

The discovery of the possible link between biological mechanisms responsible for the changes in body fat distribution, in both males and females, from childhood to adolescence, as well as the incidence of NAFLD, seems to be a key factor deserving of further attention so as to improve the prevention and treatment of NAFLD in clinical and epidemiological settings.

Despite these important findings which indicate that body fat in the trunk area is associated with NAFLD, especially in males, and that females seems to be more sensitive to develop NAFLD with the same amount of intra-abdominal fat them males. The main limitation of the present study were the use of ultrasound as the only method for diagnosis of NAFLD, without confirmation by liver function enzymes.

## Conclusion

In conclusion, the higher concentration of trunk fat and intra-abdominal fat, in obese adolescents may indicate potential to elevate the risk of developing nonalcoholic fat liver disease even when the results were adjusted for sex, age, pubertal stage and total body fat mass. Further investigations are necessary to verify the possible link between the mechanisms responsible for changes in hormonal and body fat distribution, from childhood to adolescence that may be responsible for the observed differences in the risk of higher %TF and IAF, in males and females, respectively, in the development of NAFLD.

## Abbreviations

NAFLD: Nonalcoholic fatty liver disease; SD: Standard deviation; BMI: Body Mass Index; GL: Glucose; TG: Triglycerides; TC: Total cholesterol; HDL-C: High density lipoprotein-cholesterol; LDL-C: Low density lipoprotein-cholesterol; SCF: Subcutaneous abdominal fat tissue thickness; IAF: Intra-abdominal fat tissue thickness; %TF: Percentage of trunk fat; FM: Fat mass; FFM: Fat-free mass; TFM: Trunk fat mass; SBP: Systolic blood pressure; DBP: Diastolic blood pressure

## Competing interests

The authors declare that they have no competing interests.

## Authors’ contribution

PAM: (1) conception and design of the study, (2) acquisition, analysis and interpretation of data, (3) draft of the article and selection of manuscripts to discuss the results, JM, LSS, SAU, BMMA: (1) Acquisition, analysis and interpretation of data, (2) draft of the article and selection of manuscripts to discuss the results, RAF and IFFJ: (1) conception and design of the study (2) review and approval of the final version to be submitted. All authors read and approved the final manuscript.
